# Analysis of DNA Methylation in Various Swine Tissues

**DOI:** 10.1371/journal.pone.0016229

**Published:** 2011-01-21

**Authors:** Chun Yang, Mingjun Zhang, Weiping Niu, Runjun Yang, Yonghong Zhang, Zhengyan Qiu, Boxing Sun, Zhihui Zhao

**Affiliations:** College of Animal Science and Veterinary Medicine, Jilin University, Changchun, China; New England Biolabs, Inc., United States

## Abstract

DNA methylation is known to play an important role in regulating gene expression during biological development and tissue differentiation in eukaryotes. In this study, we used the fluorescence-labeled methylation-sensitive amplified polymorphism (F-MSAP) method to assess the extent and pattern of cytosine methylation in muscle, heart, liver, spleen, lung, kidney and stomach from the swine strain Laiwu, and we also examined specific methylation patterns in the seven tissues. In total, 96,371 fragments, each representing a recognition site cleaved by either or both EcoRI + HpaII and EcoRI + MspI, the HpaII and MspI are isoschizomeric enzymes, were amplified using 16 pairs of selective primers. A total of 50,094 sites were found to be methylated at cytosines in seven tissues. The incidence of DNA methylation was approximately 53.99% in muscle, 51.24% in the heart, 50.18% in the liver, 53.31% in the spleen, 51.97% in the lung, 51.15% in the kidney and 53.39% in the stomach, as revealed by the incidence of differential digestion. Additionally, differences in DNA methylation levels imply that such variations may be related to specific gene expression during tissue differentiation, growth and development. Three types of bands were generated in the F-MSAP profile, the total numbers of these three types of bands in the seven tissues were 46,277, 24,801 and 25,293, respectively.

In addition, different methylation patterns were observed in seven tissues from pig, and almost all of the methylation patterns detected by F-MSAP could be confirmed by Southern analysis using the isolated amplified fragments as probes. The results clearly demonstrated that the F-MSAP technique can be adapted for use in large-scale DNA methylation detection in the pig genome.

## Introduction

DNA methylation is one of the main epigenetic modification mechanisms in eukaryotic organisms, playing a crucial role in the regulation of gene expression [1]–[3]. It entails the transfer of methyl groups from S-adenosine-L-methionine to cytosines and adenines by DNA methyltransferases following DNA duplication. In living cells this process has been reported as one of the most common covalent modifications. Many studies have shown that DNA methylation, especially methylation of cytosine, has been implicated in gene expression [4], [5], genomic imprinting [6], X chromosome inactivation [7], determination of chromatin structure [8], disease [9]–[11] and cancer development [12].

Methylation of CpG dinucleotides in the 5′ regulatory regions of genes often results in transcriptional inactivation, and some studies have shown that actively transcribed sequences are often methylated less than promoters and certain coding regions of silent genes [13]. Significant differences in the levels or pattern of cytosine methylation have been observed in various tissues or under different functional states in the same tissue [14]–[16]. Several studies suggested that various levels of DNA methylation may regulate tissue-specific transcription [17] and be important for normal development or differentiation [18]. But how methylation regulates gene expression is still unclear [19]. Therefore, the detection and analysis of levels and patterns of genome-wide methylation in various tissues, is essential for understanding associations between tissue-specific methylation and tissue-specific gene expression.

The methylation-sensitive amplification polymorphism (MSAP) technique is a relatively new modification of the amplification fragment length polymorphism (AFLP) technique; MSAP was first developed to determine DNA methylation events in dimorphic fungi [20]. The MSAP technique utilizes the restriction isoschizomer pair HpaII and MspI [20] (instead of MseI as in the original protocol [21]). These enzymes recognize the same restriction site (CCGG) but have different sensitivities to certain methylation states of cytosines. HpaII is inactive if one or both cytosines are fully methylated (both strands are methylated) but cleaves the hemimethylated sequence (only one DNA strand is methylated), whereas MspI is inactive if the external cytosine is fully or-hemimethylated (only one DNA strand is methylated) [22]. Thus, for a given DNA sample, the full methylation of the internal cytosines and hemimethylation of the external cytosines at the assayed CCGG sites can be unequivocally distinguished [20], [23] using these two restriction enzymes. However, it should be noted that the methylation percentages calculated by MSAP are lower than the total absolute values at the CCGG sites [20], [23], [24] since HpaII and MspI cannot distinguish several other states of the CCGG sites, including unmethylated CCGG, full methylation of both cytosine sites (^m^C^m^CGG), or hemimethylation of the internal cytosine site (C^m^CGG). Currently, MSAP is extensively applied in many areas to explore the association between methylation and plant phenotypic instability under various induced conditions [25], [26], the abnormality of cultured plants and cloned animals [27], [28], and performances of the hybrids[19], [29].

MSAP requires the use of a radiolabeled substrate, which can be harmful to human health, and its reaction products need to be analyzed by gel electrophoresis followed by X-ray film detection. This is a sophisticated approach because it requires frequent preparation of freshly labeled substrate during the experiment [19], [29], [30]. In this study, we screened and investigated the cytosine methylation of CCGG sites in the DNA of muscle, heart, liver, spleen, lung, kidney and stomach from ten Laiwu pigs by F-MSAP. This technique is a new modification of the methylation-sensitive amplification polymorphism (MSAP) technique, in which selective amplification is performed with fluorescently labeled primers instead of radiolabeled primers. We used GeneScan analysis software, an internal lane size standard, and an ABI PRISM 377 DNA sequencer.

The F-MSAP system consists of four major parts: digestion and ligation reactions, preamplification, selective amplification reactions and detection reactions.

The extent and patterns of methylation were defined, and the degree of methylation at CCGG sites was assessed and compared in all seven tissues. Furthermore, we also isolated, sequenced and verified some fragments that are differentially methylated among different tissues, which may be useful for further investigations into how methylation functions to regulate gene expression.

## Materials and Methods

### Ethics statement

Animal experiments were done in accordance with the guidelines on animal care and use established by the Jilin University Animal Care and Use Committee.

### Animal materials and DNA preparation

Tissue from muscle, heart, liver, spleen, lung, kidney and stomach were collected separately from ten Laiwu pigs (Shan Dong Agricultural University). Samples of 300 mg of each tissue were quickly washed and incubated in 900 uL lysis buffer (50 mM Tris-Cl pH 8.0, 10 mM EDTA, 100 mM NaCl, 1% SDS), and 0.1 mg proteinase K (Promega, Madison, WI, USA) with shaking for 16 h at 55°C. The genomic DNA was separated by two rounds of phenol-chloroform extraction, and traces of phenol were removed with chloroform. Genomic DNA was precipitated with sodium acetate/isopropanol followed by washing with 75% ethanol, and it was then dissolved in TE buffer (10 mM Tris-Cl pH 8.0) and stored at −20°C.

### Fluorescence-labeled methylation-sensitive amplified polymorphism (F-MSAP) assay

In the F-MSAP system, the isoschizomers HpaII and MspI, which recognize the same sequences but differ with respect to their sensitivities to the methylation of the recognition site, were used to digest genomic DNA, followed by ligation of the digested DNA to adaptors and selective amplification with fluorescently labeled primers. Then, methylation-sensitive polymorphic fragments were generated and detected using 4% denaturing PAGE run on an ABI 377 DNA sequencer (Applied Biosystems, Foster, CA, USA). DNA methylation polymorphism profiles were subsequently obtained using GeneScan 3.1 software.

The EcoRI adapter and primer have been as described by Reyna Lopez et al. [20], and the HpaII/MspI adapter and primer have been as described by Xu et al.; these primers and adapters were used with some modifications [31]. EcoRI and HpaII/MspI primers with three additional selective nucleotides were used (Table 1). All adapters and primers were synthesized by SANGON (Shanghai, China).

**Table 1:** Sequences of adapters and primers used in F-MASP.

Primers/adapters	Sequence (5′-3′)
EcoRI adapter	5′-CTCGTAGACTCGTACC-3′ 3′-CATCTGAGCATGGTTAA-5′
E+1 primers (PreAmp)	5′-GACTGCGTACCAATTC+A-3′
E+3 primers	5′-GACTGCGTACCAATTC+AAC-3′ 5′-GACTGCGTACCAATTC+AAG-3′ 5′-GACTGCGTACCAATTC+ACA-3′ 5′-GACTGCGTACCAATTC+AGT-3′ 5′-GACTGCGTACCAATTC+ATC-3′ 5′-GACTGCGTACCAATTC+ACT-3′ 5′-GACTGCGTACCAATTC+AGA-3′ 5′-GACTGCGTACCAATTC+ATG-3′
HpaII*/*MspI adapter	5′-GACGATGAGTCTAGAA-3′ 3′-CTACTCAGATCTTGC-5′
HM+1 primers (PreAmp)	5′-GATGAGTCTAGAA 1 CGG+T-3′
HM+3 primers	5′-FAM^2^-GATGAGTCTAGAACGG+TAC-3′5′-FAM -GATGAGTCTAGAACGG+TAG-3′

1 Bold indicates a core sequence that is compatible with the adapter.

2 Primer was labeled with the blue fluorescent dye 5-FAM (5-carboxyfluorescein).

DNA samples were separately digested with EcoRI/HpaII and EcoRI/MspI (TaKaRa, Dalian, China). To minimize discrepancies caused by experimental factors, digestion and ligation were performed simultaneously. The digestion-ligation reaction was performed in a volume of 25 uL containing 500 ng DNA template, 3 U EcoRI, 3 U HpaII (or MspI), 1.5 U T4 DNA ligase (TaKaRa, Dalian, China), 5 pmol EcoRI adapter, 50 pmol HpaII/*Msp*I adapter and 2.5 uL 10× T4 ligase buffer. The mixture was incubated at 37°C overnight, inactivated at 65°C for 10 min and stored at −20°C.

Preamplified PCR reactions were performed in a final volume of 20 uL containing 2 uL of ligation products, 40 ng of E+1 and H-M+1 preamplified primers (Table 1), 0.1 uL of *Ex* Taq polymerase, 1.6 uL of dNTPs (2.5 mM), 1.2 uL of MgCl_2_ (25 mM), 2 uL of 10× PCR buffer and 14.1 uL of sterile distilled H_2_O. The PCR conditions were as follows: 94°C for 5 min; 30 cycles of 94°C for 30 s, 56°C for 1 min and 72°C for 1 min; and extension at 72°C for 7 min prior to selective amplification. The PCR products from the preamplification were diluted 1 to 20 (v:v) with ddH_2_O and stored at −20°C before use.

Selective amplifications were conducted in volumes of 20 uL containing 2 uL of the diluted preamplification product, 10 ng of the E+3 primer, 40 ng of the H-M+3 primer labeled with a fluorescent dye (Table 1), 0.1 uL of *Ex* Taq polymerase, 1.6 uL of dNTPs (2.5 mM), 1.2 uL of MgCl_2_ (25 mM) and 2 uL of 10× PCR buffer. The PCR amplification reactions were performed using touch-down cycles, and the conditions were as follows: 94°C for 5 min; 13 touch-down cycles of 94°C for 30 s, 65°C (subsequently reduced each cycle by 0.7°C) for 30 s and 72°C for 1 min; 23 continued cycles of 94°C for 30 s, 56°C for 30 s and 72°C for 1 min; and extension at 72°C for 7 min. The *Ex Taq* polymerase, dNTP mixture, MgCl_2_ and *Ex* Taq polymerase buffer were purchased from TaKaRa.

An aliquot of 0.5 uL of the selective amplification products was mixed 1∶1 (v:v) with loading buffer (65% deionized formamide, 10% blue dextran, 25 mM EDTA loading solution, 25% GeneScanTM -500ROXTM size standard), heated at 95°C for 5 min and quick-chilled on ice, and then the entire mixture was loaded onto a 4% denaturing Long Ranger gel and run on an ABI PRISM 377 DNA sequencer. The electrophoresis was performed at constant power, 3000 V, 45 mA and 110 W for 4.5 h at 50°C. To detected fluorescent signals with Genescan 3.1 software, and we estimated fragment sizes with Genescan 3.1 software referring to GeneScan™ -500ROX™ standard. Finally, the data analyzed by software Genescan 3.1 software were obtained in the form of Excel table. The GeneScanTM -500ROXTM standard was purchased from Orbital.

The reproducibility of each primer pair was assessed using 4% denaturing PAGE run on an ABI 377 DNA sequencer. DNA methylation polymorphism profiles were subsequently obtained using GeneScan 3.1 software. Finally, sixteen primer combinations were selected based on clear banding patterns, polymorphism and complete reproducibility between two independent DNA extractions from the muscle tissue of the same pig.

### Cloning and sequencing of MSAP fragments

To isolate the tissue-specific MSAP fragments detected by F-MSAP, the same selected amplification products were denatured, separated by electrophoresis on a Long Ranger gel and visualized by silver staining. Several fragments were excised directly from the wet polyacrylamide gels on the plate using a razor blade. The fragments were rehydrated with 50 uL of dd H2O heated at 95°C for 5 min and slowly cooled to room temperature. The tubes were centrifuged at 12,000 g for 10 min. Aliquots of 5 uL supernatant were used as templates for reamplification. PCR reactions were performed with the same primer combinations and reaction conditions as those used in selective amplification. After verification in a 2% agarose gel, the band was recovered with a gel extraction kit (Promega, Madison, USA) according to the manufacturer's instructions. Subsequently, the product was ligated into the vector pGM-T (TIANGEN, Beijing, China) and transformed into *E. coli* strain DH5α. Sequence determinations were carried out at SANGON (Shanghai, China). Homology searches and sequence analysis were performed at the public database EMBL (http://www.Ensembl.org).

### Southern hybridization analysis

Southern blotting was conducted using parts of representative cloned fragments as probes to confirm the methylation polymorphisms detected by F-MSAP. A total of 30 ug genomic DNA from each tissue was digested separately with EcoRI*-*HpaII and EcoRI-MspI, the digestion product were electrophoresed on a 0.8% agarose gel in TBE and transferred onto Hybond-N^+^ membranes (Promega). Probe labeling, transfer to membranes, fixation, hybridization and immunological detection were carried out according to the instructions of the DIG High Prime DNA Labeling and Detection Starter Kit II (Roche Applied Sciences, Mannheim, Germany). The probe was generated by PCR amplification of plasmid DNA. The PCR amplification system was the same as in the preamplification.

### Statistical Analysis

SAS v.8.02 software (SAS Institute, Cary, NC, USA) was used to analyze the significance of differences for the different methylation levels among various tissues was analyzed by the ANOVA procedure and Duncan's multiple range test.

## Results

### DNA methylation profile of various tissues

**Figure 1:**
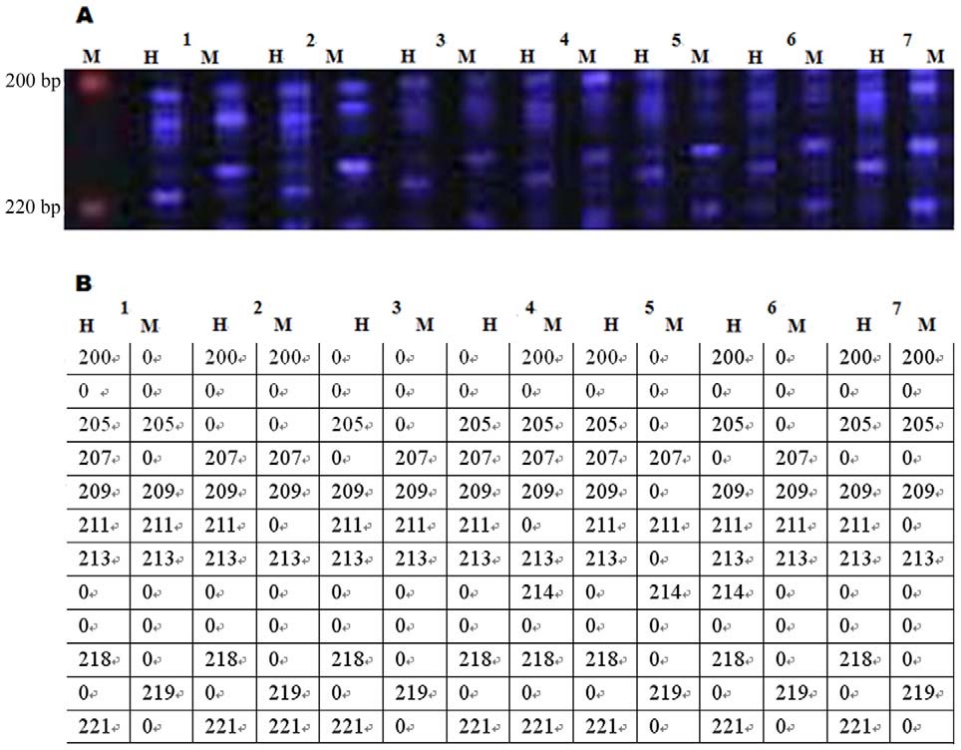
Methylation profiles of pig tissue DNA with the primer combinations H-M+TAA/E+AGT. (A) The profile from F-MSAP; (B) Data from (A) quantitated using GeneScan Analysis software; H and M refer to digestion with EcoRI/HpaII and EcoRI/MspI. Lanes 1–7 represent seven tissues, namely muscle, heart, liver, spleen, lung, kidney and stomach; M: GeneScan™ -500ROX™ size standard (represent 200 bp and 220 bp).

We used 16 pairs of selective primers, obtained from eight EcoRI primers in combination with two fluorescently labeled HpaII/MspI primers, to analyze DNA methylation at CCGG sites of various tissues from 10 pigs. The individual DNA methylation profiles for various tissues from each pig were generated by F-MSAP. Each individual genome with each primer combination is illustrated by the gel files automatically captured by the sequencer and translated by Genescan 3.1 software (Applied Biosystems). In the gel files, fluorescence represents a methylated fragment in the DNA from various tissues. A total of 96,371 fragments resolved by 16 primer pair combinations were detected by F-MSAP in DNA extracted from various tissues. For each primer combination, each individual genome displayed 35–156 fragments. In particular, fragments between 100 bp and 300 bp were highly intense, and there were few fragments exceeding 1000 bp. The F-MSAP gel file derived by GeneScan 3.1 analysis for seven tissues of an individual is shown in (Figure 1).

### DNA methylation patterns in various tissues

The isoschizomers HpaII and Msp I recognize and digest CCGG sites, but display differential sensitivity to DNA methylation. HpaII is inactive if either cytosine is fully methylated (methylation of both strands), but it cleaves the hemi-methylated sequence (only one strand methylated). MspI is sensitive only to methylation at the external cytosine and cuts in the case of inner cytosine methylation (C^m^CGG), but not in the case of outer cytosine methylation (^m^CCGG) (Table 2). Therefore, epigenetic differences can be revealed by the F-MSAP method when genomic DNA is digested and used as a template for amplification analysis to reflect the methylation status and level at the CCGG site.

**Table 2:** Methylation sensitivity and restriction pattern of isoschizomers.

Types	Methylation status	Digestibility of enzyme restriction pattern			
		HpaII	MspI	H	M
Type I	CCGGC**C**GG GGCCGGCC	Active	Active	+	+
Type II	**C**CGG GGCC	Active	Inactive	+	-
Type III	C**C**GG GG**C**C	Inactive	Active	-	+

H and M indicate the enzyme combinations of EcoRI/HpaII and EcoRI/MspI;

- band absent; + band present. Underlined cytosine is methylated.

**Figure 2:**
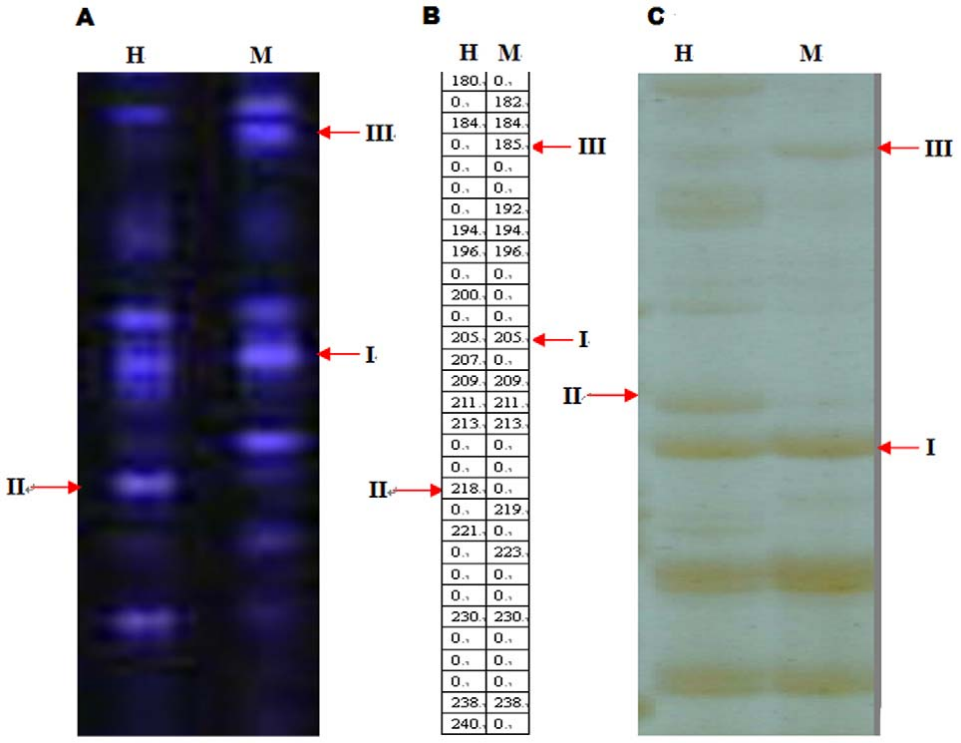
Cytosine methylation patterns with the primer combination H-M+TAA/E+AGT. (A) The profile from F-MSAP; (B) The data from (A) quantitated using GeneScan Analysis software; (C) The profile from MSAP using silver stain; H and M refer to digestion with EcoRI/HpaII and EcoRI/MspI, II and III refer to unmethylated, hemi-methylated and fully methylated sites, respectively.

In this study, the F-MSAP bands revealing methylation patterns of HpaII- and MspI- digested genomic DNA could be divided into three types of methylation status as follows (Figure 2): type I presents bands of the same length in both lanes, which indicates inner methylation of single-stranded DNA or no methylation; type II is present for HpaII but absent for MspI, which indicates outer methylation of single-stranded DNA and hemi-methylation at the outer cytosine nucleotide in the CCGG sequence; and type III is present for MspI but absent for HpaII, which indicates inner methylation of double-stranded DNA and full methylation of the CCGG sequence. In all seven tissues examined, type I was the most frequently observed at 48.1% type II is about 25.7% and type III is about 26.2%. The sum of types II and III represents the methylated fragments and accounts for about 51.9% (Table 3).

**Table 3:** Methylation levels of seven tissues in pig.

Types	Tissues						
	Muscles	Heart	Liver	Spleen	Lung	Kidney	Stomach
I	6756	6898	6629	6348	6454	6839	6336
II	4028	3574	3049	3646	3217	3570	3717
III	3926	3628	3568	3476	3660	3554	3481
Total amplified bands	14710	14100	13246	13506	13312	13963	13534
Total Methylated bands^1^	7954	7202	6617	7122	6877	7124	7198
Hemimethylation Ratio^2^(%)	26.99±5.65	25.55±5.98	23.06±3.48	27.48±9.93	24.50±6.70	25.40±6.05	27.66±6.54
Full methylation Ratio^3^(%)	27.0±7.66	25.68±2.84	27.12±2.66	25.83±5.95	27.47±7.10	25.75±5.40	25.73±5.09
Methylation Ratio^4^(%)	53.99±8.93	51.24±4.53	50.18±3.58	53.31±7.04	51.97±5.97	51.15±3.32	53.39±4.64

1 Total methylated bands  =  II+III;

2 Hemi-methylation ratio  =  II/I+II+III;

3 Full methylation ratio  =  III/I +II+III;

4 Methylation ratio  =  II+III/I+II+III.

### DNA methylation levels in various tissues

Sixteen pairs of primers were used to detect cytosine methylation at CCGG sites within the genome of muscle, heart, liver, spleen, lung, kidney and stomach in each pig. With each F-MSAP primer combination, there were fourteen lanes corresponding to seven different tissues, each of which were digested with one of the two enzyme combinations: EcoRI*-*HpaII or EcoRI-MspI. Sixteen pairs of selective primers produced a total of 96,371 fragments that were comprised of three types; type I had 46,277 bands, type II had 24,801 bands and type III had 25,293 bands in seven tissues. The methylation levels (type II + type III) of total fragments in muscle, heart, liver, spleen, lung, kidney and stomach were 53.99%±8.93, 51.24%±4.53, 50.18%±3.58, 53.31%±7.04, 51.97%±5.97, 51.15%±3.32, 53.39%±4.64 for each of the seven tissues, respectively, in ten pigs. The hemi-methylation levels (type II) were 26.99%±5.56, 25.55%±5.98, 23.06%±3.48, 27.48%±9.93, 24.50%±6.70, 25.40%±6.05 and 27.66%±6.54 for each of the seven tissues, respectively, in ten pigs. The full methylation levels (type III) were 27.0%±7.66, 25.68%±2.84, 27.12%±2.66, 25.83%±5.95, 27.47%±7.10, 25.75%±5.40, 25.73%±5.09for each of the seven tissues, respectively, in ten pigs.

To determine whether various tissues have different methylation levels in genome of pigs, an analysis of variance and Duncan's multiple range tests were performed. The results are summarized in (Table 3). In general, the methylation in the muscle was the highest among the tissues, and the methylation level of liver was the lowest. In the muscle, spleen, kidney and stomach, the hemi-methylation levels were higher than in heart, liver and lung. In contrast, in heart, liver and lung, the full methylation levels were higher than in muscle, spleen, kidney and stomach, but we found there were no significant differences in total methylation level, hemi-methylation level and full methylation level in various tissues (P>0.05).

### Tissue-specific differentially methylated regions (TDMs)

**Figure 3:**
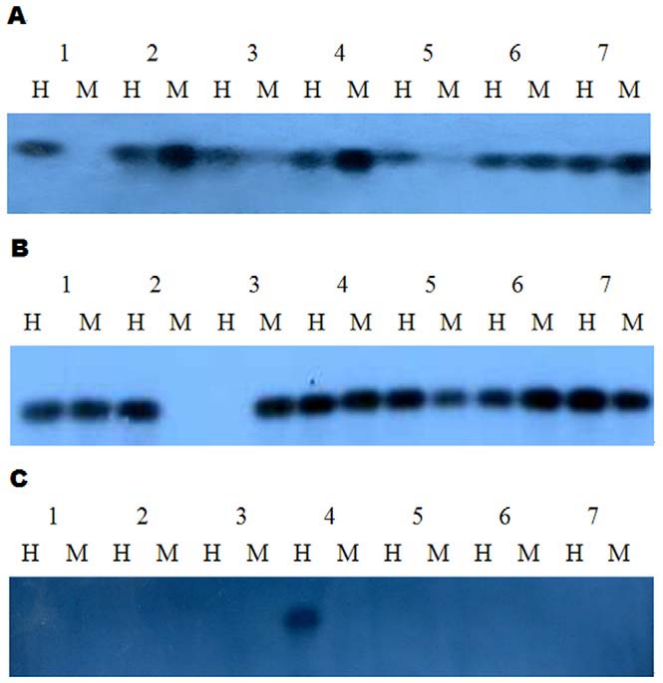
The results of Southern blots. (A) Hybridization pattern using probe T04 for the seven tissues; (B) Hybridization pattern using probe T72 for the seven tissues; (C) Hybridization pattern using probe T53 for the seven tissues; H and M indicate the enzyme combination of EcoRI/HpaII and EcoRI/MspI; Lanes 1–7 represent muscle, heart, liver, spleen, lung, kidney and stomach, respectively.

Based on previous studies and our study, tissue-specific differentially methylated regions (TDMs) include two types. Type I regions correspond to bands that were only observed in one tissue or bands that were found in six tissues but not in the seventh tissue; these bands were defined as tissue-specific methylation fragments. It is assumed that each band found only in one tissue corresponds to a methylated site that is specific to that tissue, whereas a band observed in six tissues but in the seventh tissue represents an unmethylated site specific to the seventh tissue. Type II regions correspond to bands that were observed in seven tissues but displayed different methylation patterns; these bands were defined as tissue-polymorphic methylation fragments. Some bands were found in some tissues but were missing in other tissues, and other bands were found in the EcoRI + HpaII lanes in some tissues but in the EcoRI + MspI lanes in other tissues, and vice versa; these bands displayed different methylation patterns in seven tissues. In our study, a total of 35 tissue-specific differentially methylated regions (TDMs) were found; of which, 15 TDMs were detected and displayed different TDMs types. Of the 15 TDMs, four displayed tissue-specific methylation fragments (T03, T21, T53 and T56), and eleven displayed tissue-polymorphic methylation fragments. The details are shown in Table 4, and the 15 detected fragments are shown in (Figure 3).

**Table 4:** Tissue-specific methylation patterns of CCGG sites in the genome.

Isolated fragment	Pattern of bands in F-MSAP profile													
	Muscle		Heart		Liver		Spleen		Lung		Kidney		Stomach	
	H	M	H	M	H	M	H	M	H	M	H	M	H	M
T03	+	+	+	+	+	+	+	+	-	+	+	+	+	+
T04	+	-	+	+	+	-	+	+	-	+	+	-	+	+
T05	+	-	+	+	-	+	+	+	+	+	+	+	+	+
T17	+	+	-	+	+	+	+	+	-	+	+	+	+	+
T18	+	+	-	+	+	+	-	+	-	+	+	+	+	-
T19	-	+	-	+	-	-	-	+	-	-	-	+	-	+
T21	-	-	-	-	+	-	-	-	-	-	-	-	-	-
T22	+	-	+	-	+	+	+	-	+	-	+	-	+	+
T26	-	+	+	+	-	+	+	+	+	+	+	+	+	+
T33	-	+	-	+	+	+	+	+	+	+	+	+	+	+
T36	-	+	-	+	-	+	+	+	+	+	+	+	+	+
T37	-	+	-	+	-	+	-	+	+	-	-	+	+	+
T53	-	-	-	-	-	-	+	-	-	-	-	-	-	-
T56	-	+	+	+	+	+	+	+	+	+	+	+	+	+
T72	+	+	+	-	-	+	+	+	+	+	+	+	+	+

H and M indicate the enzyme combinations of EcoRI/HpaII and EcoRI/MspI.

- band absent;+ band present.

### Southern blotting analysis

The tissue-specific differentially methylated regions (TDMs) were eluted from the silver-stained gel, reamplified and sequenced. Excised fragments from silver-stained gels were further confirmed by methylation-sensitive Southern blot analysis with the isolated fragments as probes (Figure 3). Six TDMs were missing in the silver-stained gel compared to the results from the F-MSAP profile, namely copies of these fragments appeared in the F-MSAP profile but not on the silver-stained gel. Finally, 35 fragments were obtained and compared with the EMBL database. All 35 fragments had high similarity to characterized regions of the pig genome. Sixteen of these 35 fragments were located within genes, two were in the 3′ downstream regions of genes, and four were located in the 5′ upstream regions of genes (Table 5).

**Table 5:** Sequence analysis of all methylated fragments.

Fragment	Size (bp)	Chromosome	Location	Gene	Id%
T03	100	3	5′	SNX29	100
T04	208	18	Within	JAZF1	98
T05	208	17	3′	SNF2	99
T06	259	1	No		99
T10	85	1	No		98
T13	99	2	No		100
T14	83	X	No		100
T15	99	1	Within	SFT2D1	100
T17	83	11	Within	TNFSF11	96
T18	88	4	Within	RSBN1	86
T19	120	4	Within	VAV3	99
T21	100	1	Within	ZER1	98
T22	91	4	3′	CD2	100
T24	96	4	Within	ASPH	100
T25	144	9	No		100
T26	133	14	Within	RBM2O	100
T33	249	6	Within	NHS	99
T36	129	18	Within	ELMO1	99
T37	125	6	5′	FGF21	100
T38	133	6	No		100
T40	170	6	Within	CABLES1	99
T42	88	2	Within	PCYOX1L	100
T51	123	5	Within	VWF	99
T53	137	X	Within	BACP31	99
T56	179	12	5′	LPO	100
T58	227	X	No		99
T59	247	6	No		97
T63	130	6	No		100
T64	69	3	Within	TMEM178	88
T66	99	18	No		99
T69	87	16	Within	PPAP2A	96
T71	162	15	No		98
T72	237	X	5′	KAL1	97
T73	165	15	No		100
T80	278	1	No		100

## Discussion

### Comparison between MSAP and F-MSAP

MSAP is a modified AFLP (amplified fragment length polymorphism) technique [21] to investigate cytosine methylation in genomes. In brief, isoschizomers HpaII and MspI, recognizing the same sequences but differing in their sensitivities to methylation of their recognition site, are used instead of MseI to digest the genomic DNA, then methylation sensitive polymorphic fragments can be generated after PCR amplification with compatible adapters and primer. Theoretically the development of AFLP will certainly promote the improvement of the MSAP. Huang and Sun [32] adopted fluorescent lableing in 1999 to improve AFLP system, obtained higher differentiability and detected 10%–30% more polymorphism than the conventional system. Other experiments [33], [34], also proved that fluorescent labeling system is more sensitive, safer and more practical than other detection methods. The same idea was employed in the present study to improve MSAP.

Compared to MSAP method, F-MSAP method has the following advantages. Selective amplification using fluorescence-labelled primers, in combination with gel electrophoresis using automated DNA sequencer, greatly increases the resolution and detection of the amplified fragments. Clear separation of bands can be achieved because the migration distance of each fragment is not limited by the gel size. This approach, without the need of radioisotopes or silver staining, is safe and fast. The method also increases the flexibility of laboratory work flow. Not constrained by the decay period of radioactivity, we were able to perform selective PCR amplification and/or run gels at any time that was convenient. The total time required to complete our AFLP analysis of a sample is reduced by half compared to MSAP procedure. The high number of bands generated by automated DNA analyzer indicates the nearly all amplified fragments can be detected by this method, due to better band separation by the DNA sequencing gel. The output of fragment patterns, as sharp laser prints, is more accurate and easier to interpret than X-ray films of silver-stained gels, which often show faint or non-scorable bands, especially when the number of amplified fragments is large. Thus, the non-isotopic MSAP with automated DNA sequencer detection procedure, would be preferable for safety, efficiency and high resolution to previously described MSAP detection method [20].

MSAP is a modified AFLP technique in which the isoschizomers HpaII and MspI recognize the same sequences but differ in their sensitivities to methylation of their recognition site and are used instead of MseI alone in this modified AFLP technique. The MSAP technique has been used in various studies on cytosine methylation in plant genomes and has proven to be a highly efficient and powerful tool for investigating DNA methylation in plants [19], [29], [35]–[38]. F-MSAP was established by Xu Qing [39] and has been only used to analyze DNA methylation of various tissues in chicken [40], where the MSAP and fluorescence system is a practical assay for investigation of DNA methylation patterns. The F-MSAP has the main advantages of safety, time efficiency, high sensitivity and ease of automation over standard MSAP. For the first time, in the present study we use this system to investigate DNA methylation patterns in the genome of tissues from adult healthy pigs. The results clearly demonstrate that F-MSAP allows efficient, high-throughput detection of cytosine methylation of the whole genome.

However, this technique also has four major constraints associated with its resolving power. First, it depends on template DNA quality. Second, this method can only detect a limited spectrum of bands [21]. Third, this method can only investigate a small proportion of cytosines in the genome because the isoschizomers used only recognize CCGG sites in the genome. Fourth, the types of non-methylation and inner-methylation of a single strand cannot be distinguished because both HpaII and MspI are capable of recognizing the sites of non-methylation and inner-methylation of a single strand [38]. Both the HpaII and MspI enzymes recognize CCGG sites [22], and in many cases methylation of cytosine residues in the restriction sites could not be detected between the two digestions, such as the occurrence of methylation of all four cytosine residues at the recognition site. For these reasons, the results of F-MSAP analysis possibly underestimate the actual levels of methylation in the genome and miss some tissue-specific methylation fragments.

In this study we use the F-MSAP method to compare the levels of DNA cytosine methylation in muscle, heart, liver, spleen, lung, kidney and stomach from pigs for the first time. The results show that there are different methylation levels and patterns in various tissues, but, the methylation levels in various tissues are no significant differences (P>0.05). First, in our study the hemi-methylation sites (type II) occur more frequently than full methylation sites (type III) in the muscle, spleen, kidney and stomach. These results are consistent with the chicken [39], [40]. Second, the full methylation sites (type III) occur more frequently than hemi-methylation sites (type II) in the heart, liver and lung. These results are consistent with plants [21], [35], [38]. The different DNA methylation levels and patterns in various tissues are possibly related to the regulatory mechanism of gene expression during tissue differentiation and developmental processes [38], [39], [41]. Different species and genetic backgrounds or different methods of methylation detection are likely to exhibit different DNA methylation levels and patterns. Moreover, different tissues within the same species may also exhibit differences in methylation [41]. The function of methylation differences are complex and largely unknown. Compared to MSAP, the different labeling methods for the primers applied in F-MSAP may contribute to this difference. In summary, the observed differences in DNA methylation levels and differences in DNA patterns in the seven tissues may play roles in the process of cell differentiation, development and corresponding gene expression in these seven tissues.

Previous studies have demonstrated that some genes were characterized by their variable levels of methylation in different tissues, and undermethylation in these genes in general correlated with tissue-specific gene expression [16], [41], [42]. However, there are some studies expressing doubts on the correlation between expression and undermethylation [43]. To date, major studies regarding methylation have all focused on the promoter regions of genes and found that, if CpG islands in this region were hypermethylated or demethylated, the gene would likely be correspondingly silenced or activated [44]. In some cancer studies, aberrant epigenetic modifications such as hypermethylation or demethylation can change the expression of genes [45]. However, it should be noted that cytosine-methylated 5-CCGG-3 sequences are distributed in repetitive sequences in the coding and noncoding regions that contain introns, repetitive elements and potentially active transposable elements [46]. In this study, we found that among 35 sites of TDMs, the sequence ranged from 88 to 278 bp. Sixteen are located within introns while four are located in the 5′ upstream regions, and two are located in the 3′ downstream regions of genes. Moreover because the pig genome is not complete, there are 13 sites that we cannot locate in genes, but can only locate at the chromosome level. Because the number of tissues is large, we rarely found a band present only in one tissue, but we often found bands present in two or three tissues or bands present in different patterns in different tissues. To verify whether the tissue-specific methylation found in this study is related to expression of the corresponding genes, further studies are needed.

### Conclusion

In this study we used F-MSAP that is a modified MSAP technique [20] to investigate cytosine methylation in genomes and found different methylation levels at CCGG sites in the genome of seven tissues from pigs.The results clearly demonstrated that F-MSAP is highly efficient for large-scale detection of cytosine methylation and can be further extended to research on genome of other animals and plants that have complex genome and are rich in methylation polymorphism. Based on the results from this study and the functions during the gene expression, we think that differences in methylation levels and patterns may be important to regulate tissue-specific gene expression and cell differentiation, however we need to perform further studies to confirm this.
